# Extraction of Teeth

**Published:** 1853-04

**Authors:** J. Taylor


*Extraction of Teeth.* By Dr. J. TAYLOR. [CONTINUED.]

In our last, we made a few remarks on the key, its use, mode of application, &c. &c. We shall now give our views of the forcep. This instrument for common application possesses advantages far superior to any other now in use, and we see no principle by which the application of force can be so judiciously applied. Hence the proper construction of this instrument is of great importance to the profession.

It might be a difficult matter to trace back the origin of this instrument. The leaden pair found in the temple of Apollo could hardly have been a model left in the patent office—nor could they have been made for use—for such forceps would prove very inefficient. They appear, however, to have been in use at an early period, but within our recollection they were universally badly adapted for the use designed.

The recent improvements in this instrument appear to have been first started by Mr. Cartwright, of London, and Mr. Snell of this country. Since their day, we presume, that most of our old and experienced operators have had a hand in these improvements. The instrument in every particular has been improved in the last ten years. For what was then considered a good set of forceps, would now pretty generally be rejected.

There are two or three general requisites which this instrument should always have, and without which we regard them as very faulty. The first is adaptation to the teeth, and this means a great deal. It does not mean merely a fit to the teeth out of the mouth, neither does it mean a fit when applied to the teeth in the mouth, which will place the operator in an awkward or constrained position, or which will place the operator directly in front of the patient. A position which does not give free motion to the flexor muscles of the arm used in handling the forcep, is a bad one. We wish to *draw* the teeth with this instrument, and not *push* them. We use our right hand, when operating with the forcep, and stand at the right side of our patient, back of his arm. This is our position in extracting all of the teeth. In operating on the lower teeth, we are a little farther back, or nearly behind the patient, and standing on a stool, so that we can stoop over the right shoulder, and look into the mouth. The first and great advantage of this position is that we are out of the reach of our patient, and second, we can hold more securely the head. In extracting the under teeth, our left arm passes over the left shoulder, and we lay hold of the patient's chin with that hand. The chin resting in the palm of the hand.

This enables us to give stability to the lower jaw and prevents luxation, &c. Without the head is held steady, it is impossible to apply the right force for the extraction of the tooth—hundreds of frail teeth are broken from this cause.

A forcep which would place us in front and to the left of our patient, for the removal of the left molars in the inferior maxilla, we regard as very objectionable, because we cannot so well steady the head, and the patient may and often will involuntarily throw up both hands to push us away, and will also often attempt to jerk the head from our grasp. True, if we have hold of a firm and strong tooth, in this he may fail, but if the tooth is frail, it is very often broken.

To give us the position we have stated, it will be seen that a very different kind of forcep will be required for either side of the mouth.

The next general requisition in these instruments, is what we would call perfect adaptation to the tooth. This was, until the last few years, the great defect of this instrument, for had the forcep been only properly adapted to one class of teeth, their excellence would have been apparent, and this would have led to different forms for the different teeth. A forcep which in grasping the neck of the tooth would press hard on the crown, would not answer, except, first, to crush the crown, and then remove the roots. The old blades made, serrated on their inner surface, to prevent them from slipping, is far more preposterous than the advice of ———, which was, to shake them well before extracting, for the latter is often very necessary, the former never. We had rather have the blades so constructed as to *slip up* on the neck of the teeth. The easier they pass under the gum the better, requiring less force to get them where they should generally be.

The adaptation should be such as to embrace the tooth, above the free margin of the gum, and indeed, permit the points of the forcep blades to come in contact with the alveolus, without undue pressure upon the crown. This can be accomplished in almost all cases—and for this purpose, the points of the blades should be brought to knife-like points. This will require that the blades be finely tempered; not so hard and brittle as to break, and not so soft as to bend under the force applied. The inner surface of the blades should be hollowed out to fit the crown and neck of the teeth, like a mould, allowing the hardest pressure to be made at, or near the points of the blades, where they embrace the most sound and firm portion of the tooth.

The third requisition, and of some consequence, although, not so essential as the others is, that the handle be adapted to the hand. When applied to the tooth, the hand should not be forced too much open; this would prevent a secure hold, and if the handles were too smooth they would slip in the hand, and if made with sharp angles, they would hurt the hand. They should fill up the hand so as to secure a firm grasp, and that portion embraced by the palm, be roughened, to prevent slipping—and stiff enough to prevent springing. In our large forceps, for the extraction of the molars, and indeed, the bicuspids, we have one blade of the handle to curve around the little finger. This gives additional security when much force is required.

From the adaptation here described, it will be seen, that four or five, or even six or eight pair, will not meet all the cases, or be adapted to all the teeth—one pair of forceps may answer the superior inscisors, the cuspidati, and bicuspides; yet, there are lateral inscisors, too small for the forcep properly adapted to the others. We then, need a right and left molar for the superior teeth, and indeed, prefer two pair for either side. That for the posterior molar, a little more curved, and not so large as for the anterior; besides, the posterior molar is generally more rounded on the posterior portion of its labial face, and the forcep which fits a large well formed anterior molar, does not fit this. For teeth of this class, not much weakened by decay at the necks, we prefer the blade which embraces the labial roots, to be double pointed, to suit both roots; and for those very much decayed, we wish a hawk-bill point to pass up between the roots. The blade which applies to the palital root, should be single grooved. This arrangement would require six pair of forceps for these four teeth, and we see no reason why this number should not be had, particularly, when the operation can be facilitated thereby.

As we stand in the same position for the extraction of the molars of both sides, we have the handles of these forceps made all alike, and only change the blades so that the palital blade for the left side is adapted to the labial of the right. In the extraction of these teeth, the face of the patient is always turned towards me, and the head held with the left arm and hand. The old form of forcep, which required the patient's head to be turned from the operator, and the arm thrown from the body, with the back of the hand which holds the forcep, turned upwards, is to us, a perfectly useless instrument; because, in this constrained position, we loose the free use of the instrument. The change of this forcep to that already described, was made by ourself, some ten or twelve years since, and we believe the pattern is now most generally adopted. If in use before, it is not to our knowledge.

The forcep for the extraction of the superior dens-sapientiæ, is bent at two obtuse angles—the blades broad and single grooved; which adapts to a large majority of the teeth. These bends in the bar of the instruments, throw the handle on a line with the posterior bicuspid, or anterior molar teeth. The forcep for the superior bicuspids, cuspidati, and incisors, is slightly curved, just where the blades and handles unite with the bar of the instrument. This is to prevent pressure on the lower teeth, or what is worse, on the lip—wounding this, when caught between the bar of the instrument, and the lower teeth. We have recently, made a slight improvement in the blades of this instrument. It simply consists in lengthening and sharpening them, like a root forcep, and giving room for the crown of the tooth. The advantage is this, if the teeth are frail above the gum, your hold is as far under this, as the alveolus will permit. We have simply, a root forcep, adapted to the root before the crown is broken off. We regard this, as much more secure.

The further description of the different forceps used, and the manner of application, for the removal of the teeth, will be given in our next number.

